# Severe hypoxemia in a patient with right ventricular myocardial infarction and SARS-CoV-2 infection

**DOI:** 10.1186/s12872-022-02765-9

**Published:** 2022-07-28

**Authors:** Francisco Albuquerque, Pedro M. Lopes, Catarina Brízido, Christopher Strong, Maria João Andrade, Pedro de Araújo Gonçalves, António Tralhão

**Affiliations:** Department of Cardiology, Hospital de Santa Cruz, Centro Hospitalar Lisboa Ocidental, Av. Prof. Dr. Reinaldo dos Santos, 2790-134 Carnaxide, Lisbon Portugal

**Keywords:** Myocardial infarction, 2019-novel coronavirus pneumonia, Hypoxemia, Foramen ovale, patent, Case report

## Abstract

**Background:**

Refractory hypoxemia after right ventricular myocardial infarction and concomitant SARS-CoV-2 infection represents an uncommon, yet particularly challenging clinical scenario. We report a challenging diagnostic case of refractory hypoxemia due to right-to-left shunt highlighting contemporary challenges and pitfalls in acute cardiovascular care associated with the current COVID-19 pandemic.

**Case presentation:**

A 52-year-old patient admitted for inferior acute myocardial infarction developed rapidly worsening hypoxemia shortly after primary percutaneous coronary intervention. RT-PCR screening for SARS-CoV-2 was positive, even though the patient had no prior symptoms. A computed tomography pulmonary angiogram excluded pulmonary embolism and showed only mild interstitial pulmonary involvement of the virus. Transthoracic echocardiogram showed severe right ventricular dysfunction and significant right-to-left shunt at the atrial level after agitated saline injection. Progressive improvement of right ventricular function allowed weaning from supplementary oxygen support. Patient was latter discharged with marked symptomatic improvement.

**Conclusion:**

Refractory hypoxemia after RV myocardial infarction should be carefully addressed, even in the setting of other more common and tempting diagnoses. After exclusion of usual etiologies, right-to-left shunting at the atrial level should always be suspected, as this may avoid unnecessary and sometimes harmful interventions.

**Supplementary Information:**

The online version contains supplementary material available at 10.1186/s12872-022-02765-9.

## Background

In late 2019, infection with a new coronavirus, causing severe acute respiratory syndrome, was reported in Wuhan—China [1]. On the 3rd of November 2020, COVID-19 was characterized by the World Health Organization as a pandemic [2]. The interaction of SARS-CoV-2 virus with the cardiovascular system and its impact on the epidemiology, natural history, clinical manifestations and management of patients with acute cardiovascular disease has generated considerable interest in the scientific community [3].

Refractory hypoxemia after right ventricular myocardial infarction and concomitant SARS-CoV-2 infection represents an uncommon, yet particularly challenging clinical scenario. After exclusion of usual etiologies, right-to-left shunting at the atrial level should always be suspected as a possibility, as this may avoid unnecessary and sometimes harmful interventions. Contemporary challenges and pitfalls in acute cardiovascular care associated with the current COVID-19 pandemic are highlighted in this report.

## Case presentation

**Fig. 1:**
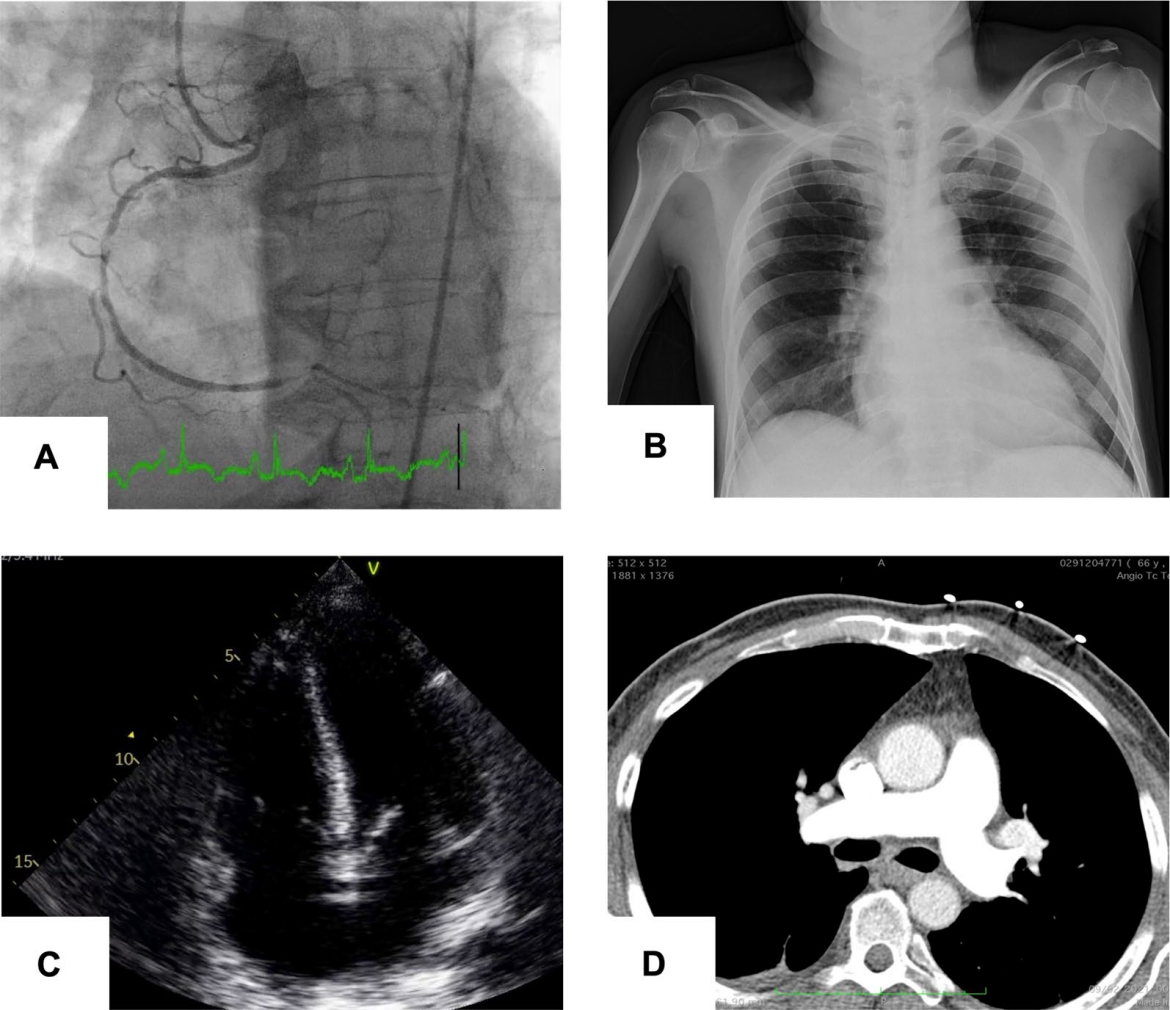
**A** Coronary angiography showing occlusion of the proximal right coronary artery. **B** Chest X-ray displaying absence of pulmonary congestion, consolidation or diffuse parenchymal infiltrate. **C** Transthoracic echocardiography on admission: apical four-chamber view showing right ventricular enlargement. **D** Computed tomography pulmonary angiogram excluding thrombus

A 52-year-old man with hypertension, insulin-treated type 2 diabetes and dyslipidemia, was admitted to the emergency room with prolonged chest pain, nausea and general distress. Physical examination showed normothermia (36.8 °C), a blood pressure of 110/75 mmHg, oxygen saturation of 96% on room air, normal lung sounds and adequate peripheral perfusion. A 12-lead electrocardiogram (EKG) showed sinus tachycardia at 110 beats per minute and ST-segment elevation on the inferior leads (II, III and aVF). After being given a loading dose of antiplatelet therapy (aspirin 300 mg and ticagrelor 180 mg) and a bolus dose of 70 U/Kg of unfractionated heparin, the patient was immediately transferred to the cardiac catheterization laboratory. The coronary angiography through transradial access, performed nine hours after pain onset, revealed significant three-vessel disease with a critical lesion of the proximal right coronary artery (RCA) (Fig. 1A).

Primary percutaneous coronary intervention with a drug-eluting stent was performed resulting in Thrombolysis in Myocardial Infarction (TIMI) grade III flow and resolution of the symptoms. Mandatory by institutional protocol at admission, screening for SARS-CoV-2 infection with real-time polymerase-chain reaction nasal swab was positive. He was transferred to an isolation room in the cardiac intensive care unit (CICU) for further management.

Over the first 6–8 h after admission, the patient developed worsening dyspnea with severe hypoxemia, with a PO_2_/FiO_2_ ratio < 100 mmHg, requiring oxygen supplementation with a non-rebreathing face mask (FiO_2_ ~ 100%) to maintain SpO2 90–92%. The pulmonary auscultation remained unremarkable and chest radiography displayed mild cardiomegaly and no signs of pulmonary congestion, consolidation or diffuse parenchymal infiltrate (Fig. 1B). Transthoracic echocardiogram (TTE) showed preserved left ventricular ejection fraction, inferior wall hypokinesia, a dilated right ventricle (RV) with severely reduced systolic function (TAPSE 10 mm, RV peak systolic velocity 6 cm/s) and no valvular abnormalities (Fig. 1C). The inferior vena cava was plethoric and without collapse during inspiration. To investigate the cause of the hypoxemia, a computed tomography pulmonary angiogram was performed, excluding pulmonary embolism and showing a mild bilateral crazy-paving pattern, consistent with mild pulmonary parenchymal involvement (Fig. 1D).

Despite no significant radiological evidence of SARS-CoV-2 pulmonary involvement or signs of acute left heart failure, the patient persisted with tachypnea and severe hypoxemia requiring escalation to a high-flow nasal cannula (HFNC). Given the disproportionate severity of hypoxemia, alternative causes were considered. TTE with agitated saline injection revealed a significant right-to-left interatrial shunt during spontaneous breathing (Additional file 1: Movie 1), suggestive of a *patent foramen ovale* (PFO).

PFO closure in the acute phase was considered, but after diuretic escalation with intravenous furosemide, serial echocardiographic evaluations showed progressive improvement of RV function, allowing weaning from supplementary oxygen support over the first 5 days after admission. The patient was discharged after three weeks, with a scheduled cardiac surgery consultation to consider coronary artery bypass graft surgery plus surgical closure of the PFO. A TTE and transesophageal echocardiogram (TEE) performed 3 months after the initial event revealed a PFO with tunneled morphology and bidirectional shunt (Additional file 2: Movie 2) as well as improvement of the RV function (TAPSE 16 mm; 3D Ejection Fraction 42%, peak systolic longitudinal strain of RV free wall − 19.1%) with a non-dilated cavity. After a 4-month follow-up, the patient remains asymptomatic.

## Discussion and conclusion

In the case of our patient, several possible causes for the severe hypoxemia had to be considered.

In the setting of a positive SARS-CoV-2 test, the most obvious was COVID-19 disease, which has been associated with interstitial pneumonia and severe acute respiratory distress syndrome in approximately 15% of patients [1]. Concomitant arterial and/or venous thromboembolic phenomena have also been described in patients with COVID-19 disease [1], and concurrent pulmonary embolism was also considered in the differential diagnosis. Furthermore, with the pandemic, healthcare systems faced major organizational changes resulting in delayed care delivery and an increased incidence of mechanical complications associated with acute coronary syndromes [2], which were also contemplated.

Determining the etiology of hypoxemia is crucial for appropriate clinical management. In our case, the initial differential diagnosis included pulmonary edema due to acute left heart failure, a mechanical complication of myocardial infarction, severe COVID-19 disease and pulmonary embolism. A contrast-enhanced computed tomography excluding pulmonary embolism and grading COVID-19 pulmonary disease as mild, led to consideration of alternative causes such as the presence of right-to-left shunting. TTE with agitated saline injection confirmed the clinical suspicion. In this context of right ventricular myocardial involvement in acute inferior MI, a previously asymptomatic PFO may lead to severe hypoxemia, since acute RV failure increases right-sided cardiac pressures resulting in right-to-left shunting.

Although refractory hypoxemia is a common indication for invasive mechanical ventilation (IMV), intrathoracic positive pressure would likely worsen cardiac output by decreasing right ventricular preload and increasing its afterload, precipitating hemodynamic deterioration. Moreover, augmented mean airway pressure would paradoxically decrease oxygenation by further promoting right-to-left intracardiac shunting [3]. Conversely, profound type 1 respiratory insufficiency with absent signs of increased work of breathing and disproportionate chest-CT findings commonly occurs in severe Covid-19 [4]. This conundrum has sparked an intense debate on the decision on whether to start or delay IMV in these patients, given the balance between patient self-inflicted lung injury and the complications of IMV [5]. Thus, timely elucidation of the underlying mechanism through multi-modality imaging was paramount to avoid IMV, allowing RV end-diastolic wall stress reduction through diuretic therapy and myocardial recovery after revascularization to take effect. Ancillary use of HFNC further contributed to patient stabilization, by normalizing PaO2 and reducing hypoxic vasoconstriction.

However, COVID-19 infection and isolation posed specific challenges in the management of this patient, disrupting the usual routine workflow. For instance, a TEE would have been performed earlier to better characterize the intracardiac shunt before surgery. More so, complete surgical revascularization was postponed a few months, as the patient was deemed unsuitable for cardiac surgery until Covid-19 respiratory sequelae were excluded.

In our patient, percutaneous PFO closure was considered, but was later deemed unwarranted due to RV function recovery. Given that the patient had residual 2-vessel disease suitable for surgical revascularization, atrial septum inspection and closure of the PFO were proposed during follow-up.

Refractory hypoxemia after RV myocardial infarction should be carefully addressed, even in the setting of other more common and tempting diagnoses. After exclusion of usual etiologies, right-to-left shunting at the atrial level should always be suspected, as this may avoid unnecessary and sometimes harmful interventions.

## Supplementary Information

**Additional file 1. Movie 1:** Contrast transthoracic echocardiography with agitated saline injection showing significant right-to-left interatrial shunt

**Additional file 2. Movie 2:** Transesophageal (2D) echocardiography 3 months later: modified mid-esophageal view showing a patent foramen ovale with tunneled morphology and bidirectional shunt

## Data Availability

The authors declare that all other data supporting the findings of this study are available within the article and its Additional files 1 and 2.

## Abbreviations

CICU: Cardiac intensive care unit
EKG: Electrocardiogram
HFNC: High-flow nasal cannula
PFO: Patent foramen ovale
RCA: Right coronary artery
RV: Right ventricle
TAPSE: Tricuspid annular plane systolic excursion
TIMI: Thrombolysis in myocardial infarction
TTE: Transthoracic echocardiography
STEMI: ST-segment elevation myocardial infarction
